# Harnessing Dynamic Supramolecular Interactions for Lanthanide Detection via Computational Pattern Recognition of Magnetic Resonance Fingerprints

**DOI:** 10.1021/jacs.5c03583

**Published:** 2025-05-19

**Authors:** Elad Goren, Balamurugan Subramani, Liat Avram, Alla H. Falkovich, Or Perlman, Amnon Bar-Shir

**Affiliations:** † Department of Molecular Chemistry and Materials Science, 34976 Weizmann Institute of Science, Rehovot 7610001, Israel; ‡ Department of Chemical Research Support, Weizmann Institute of Science, Rehovot 7610001, Israel; § Department of Biomedical Engineering, Tel Aviv University, Tel Aviv 6997801, Israel; ∥ Sagol School of Neuroscience, Tel Aviv University, Tel Aviv 6997801, Israel

## Abstract

The reliance of modern technology growth on lanthanides presents dual challenges: securing sustainable sources from natural or recycled materials and reducing environmental harm from waste discharge. However, the similar ionic radii, oxidation states, and binding affinities of Ln^3+^ ions hinder their nondestructive detection in mixtures. Furthermore, the overlap of spectroscopic signals and the inapplicability for opaque solutions limit the harness of luminescent sensors for differentiating one Ln^3+^ from another. Here, we introduce ^19^F-paramagnetic guest exchange saturation transfer magnetic resonance fingerprinting (^19^F-paraGEST MRF), a rapid signal acquisition, encoding, and analysis approach for detecting specific Ln^3+^ in mixtures. Based on a small-sized experimental ^19^F-paraGEST data set, we generated a de novo dictionary of ∼2500 combinations of Ln^3+^ mixtures, resulting in ∼7,000,000 simulated ^19^F-paraGEST MRF patterns of different Ln^3+^ concentrations. This dictionary was later used for computational pattern recognition of experimental NMR signal evolutions (“fingerprints”), utilizing a rapid computational approach executable on a standard laptop within seconds. Hence, fast and reliable multiplexed lanthanide detection in complex mixtures was enabled. Demonstrated through the analysis of lanthanides’ content of permanent magnets from a hard disk drive, this MR-based method paves the way for broader applications of lanthanide detection in murky, nontransparent mixtures and further exploration of supramolecular sensors in diverse scenarios.

## Graphical abstract

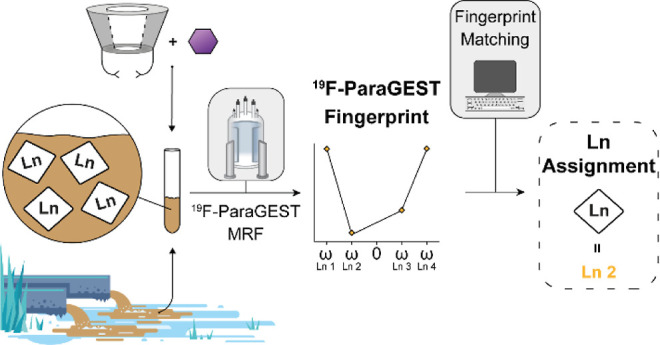

## Introduction

The unique optical, magnetic, and electronic properties of lanthanide ions (Ln^3+^) make them attractive for various fields of scientific research and modern technology. For example, lanthanides are integral components of several types of catalysts in chemical reactions^1–3^ and essential structural and functional elements in various nanoformulations.^4,5^ They are pivotal for luminescent materials,^5^ permanent magnets,^6^ electrodes, and electrolytes in rechargeable batteries,^7,8^ contrast agents used for medical imaging,^9,10^ and related multiplexed tagging applications.^11,12^ The excessive use of lanthanides in the modern age, resulting in a growing global demand, underscores the need for the location of additional lanthanide sources. A detection method is required to discover new natural sources or to evaluate the lanthanide content of recyclable materials to ensure a sustainable supply.^13,14^ Simultaneously, the harmful environmental impact of lanthanides, particularly from electronic and industrial wastes,^15,16^ necessitates robust sensing capabilities to identify and quantify them in complex, multicomponent solutions.

Luminescence-based techniques, particularly those employing fluorescence signals, have emerged as platforms for detecting lanthanides in complex mixtures and waste materials.^17^ Some of these methods capitalize on the unique photophysical properties of lanthanides, such as their sharp emission lines and long-lived excited states, to achieve high sensitivity and selectivity.^18^ Additionally, organic fluorescent ligands and polymers have been developed to coordinate lanthanides and enhance their luminescence through antenna effects and energy transfer.^19–24^ Similarly, fluorescent lanthanide-binding proteins were engineered for optical sensing of Ln^3+^ ions in aqueous solutions.^25–28^ Applied in environmental monitoring, recycling, and industrial processes, these approaches offer nondestructive and real-time analysis capabilities. However, significant challenges remain for luminescence-based sensors, including distinguishing between chemically similar lanthanide ions and the inability to produce signals in murky and turbid solutions. These challenges call for advanced nonluminescent detection systems that can provide high spectral resolution in lanthanide mixtures, overcoming their chemical similarity.

Magnetic resonance fingerprinting (MRF) is an increasingly investigated signal acquisition and reconstruction paradigm, which utilizes the unique, multidimensional signal characteristics of various molecular environments to achieve precise and quantitative measurements in both NMR and MRI setups.^29–31^ A specialized form of MRF is chemical exchange saturation transfer (CEST)-MRF,^32,33^ which focuses on quantifying the chemical exchange processes of exchangeable protons with bulk water. CEST-MRF generates a unique encoding for different metabolite concentrations and proton exchange rates in a complex biological milieu by adopting a pseudorandom pulse sequence, which varies several saturation parameters across repeated and short signal acquisitions.^34^ To decode the experimentally measured data, the underlying biophysical theory (as represented by the Bloch–McConnell equations)^35^ is harnessed to generate a simulated signal dictionary of various signal manifestations. This dictionary is then compared with the experimental data via machine learning or pattern-matching algorithms^36–40^ to determine the proton exchange properties of the compound of interest. Recent preclinical and clinical studies have demonstrated the potential of CEST-MRF as a rapid and quantitative means for detecting subtle biological variations that would be challenging to discern using traditional MR techniques.^34,41^

The recent implementation of CEST-NMR principles to study supramolecular systems by exploiting the dynamic nature of host–guest complexes has opened opportunities for better characterization and a wider range of applications of host–guest systems.^42,43^ Specifically, the use of fluorinated organic guests to benefit from background-free ^19^F-NMR and ^19^F-MRI signals and ^19^F-guest exchange saturation transfer (^19^F-GEST) has been profitable for studying and quantifying dynamic host–guest processes^44–48^ and for in vivo molecular imaging.^49^ Adapting lanthanide-modified molecular hosts, specifically α- and β-cyclodextrins (Ln-CDs),^48^ the pseudocontact shift (PCS) property of paramagnetic lanthanides was used to modulate the ^19^F-MR chemical shift offset of a given fluorinated guest. The array of ^19^F-paraGEST chemical shifts, characteristic of each Ln-α-CDs, was used for generating multiplexed MRI maps.^50^ Applying CEST-MRF principles for ^19^F-paraGEST NMR, we developed an approach for lanthanide sensing in various mixtures. By combining a rapid acquisition scheme with a de novo synthesized dictionary of simulated ^19^F-paraGEST signal evolution fingerprints and computational pattern matching, we have obtained the specific detection of multiple lanthanides in mixtures, including those extracted from an electronic device.

## Results and Discussion

Figure 1 schematically demonstrates the basic principles of the proposed approach for sensing lanthanides in mixtures based on their ^19^F-paraGEST MRF. In an aqueous solution, the molecular host α-CD-DTPA readily binds neighboring Ln^3+^ ions (Figure 1a) to obtain a mixture of different Ln-α-CD hosts (Figure 1b). Upon the addition of a fluorinated guest, which exists in a dynamic exchange between its free and Ln-α-CD-bound states (Figure 1c), a characteristic z-spectrum is expected in a ^19^F-paraGEST NMR experiment (Figure 1d). Such spectrum contains multiple ^19^F-paraGEST effects, each one representing a single Ln^3+^ of the obtained Ln-α-CD hosts (Figure 1c).^48,50^ Directly assigning the ^19^F-paraGEST effects from a z-spectrum to the corresponding lanthanides may be challenging, especially when these effects show overlapped chemical shift offsets (i.e., Δω′s). Moreover, to collect a z-spectrum with the desired spectral resolution for identifying all ^19^F-paraGEST peaks, a data set that spans the extensive range of Δω′s (ca. 45 ppm) must be acquired. This process results in long acquisition and postprocessing times, can lead to misassignment of the ^19^F-paraGEST effect with the corresponding Ln^3+^, and is dependent on subjective interpretation of the data.

**Figure 1:**
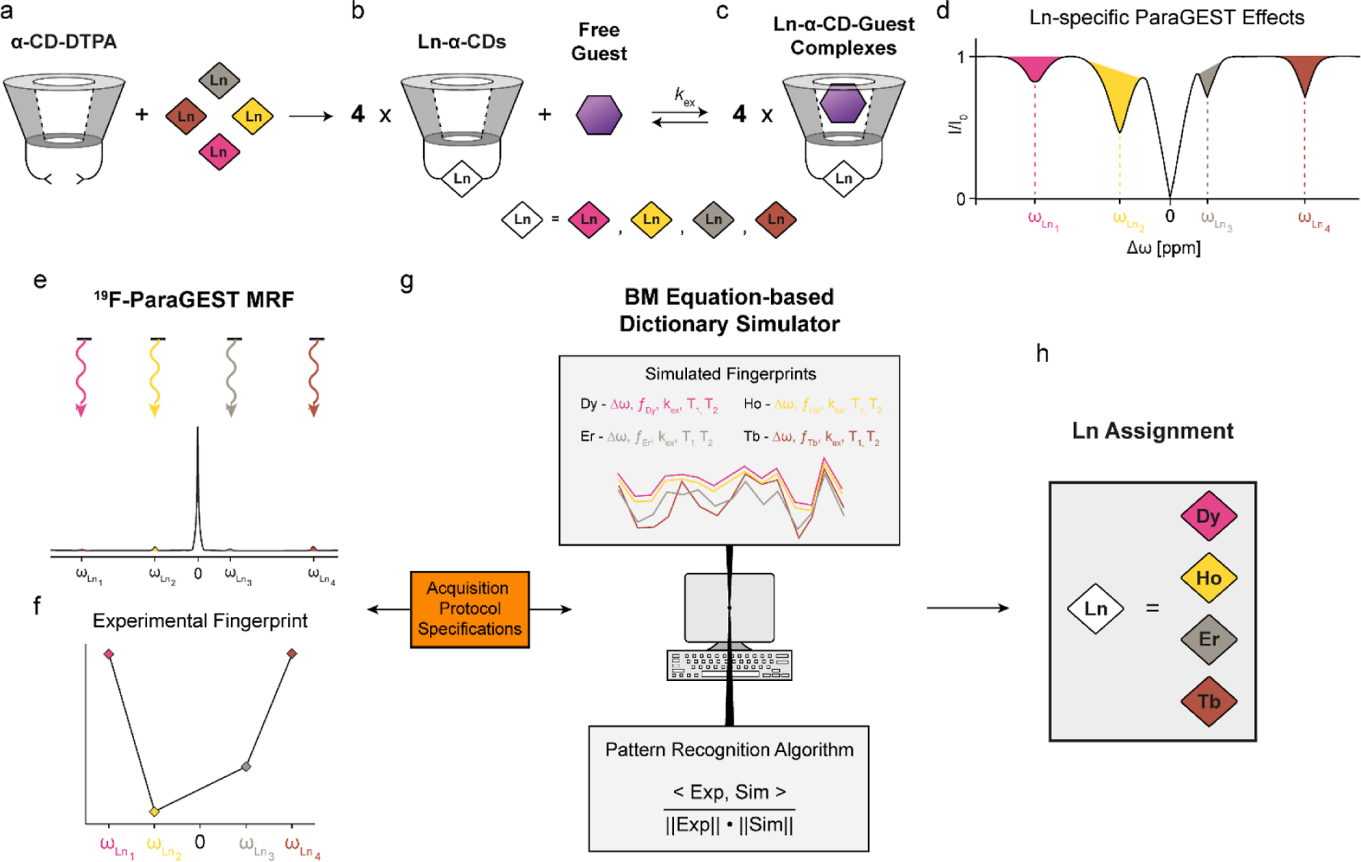
^19^F-paraGEST MRF concept illustration. Scheme describing the exchange process in a multiple host–single guest system, which is the result of (a) α-CD-DTPA chelating free lanthanides, yielding (b) Ln-α-CDs modified with different lanthanides; (c) the addition of free ^19^F-guest molecules produces different Ln-α-CD–guest complexes. Since the host–guest interactions are noncovalent, the guest molecule exchanges between the free state and the different bound states; (d) the resulting ^19^F-paraGEST z-spectrum, which requires prolonged acquisition times due the need of multiple saturation pulses at multiple frequencies; (e) ^19^F-paraGEST MRF acquisition: presaturation pulses are applied at specific frequency offsets relative to the NMR peak of the free guest; (f) the normalized signal intensity of the free guest is recorded at each saturation point, resulting in a unique ^19^F-paraGEST fingerprint; (g) assignment: the experimental fingerprint is compared to a large number of simulated fingerprints (i.e., dictionary) using a pattern recognition algorithm; and (h) the identity of the chelated lanthanide is determined using the matched simulated fingerprint.

Expanding the principles of CEST-MRF^33^ for ^19^F-paraGEST opens an opportunity to overcome these limitations by replacing the classic approach with a specific and short acquisition protocol (Figure 1e). Applying the rapid acquisition protocol results in a characteristic signal trajectory (Figure 1f, termed ^19^F-paraGEST fingerprint) for each Ln-α-CD. This approach shortens the data acquisition time and offers a simulated “dictionary” of ^19^F-paraGEST trajectories (Figure 1g, top) computationally generated for numerous Ln-α-CD combinations, beyond those applicable for experimental data collection. Using a pattern recognition algorithm (Figure 1g, bottom) to compare experimentally measured ^19^F-paraGEST MRF trajectories (Figure 1e,f) with all simulated dictionary entries, the identity of the lanthanides present in the studied sample can be blindly determined (Figure 1h), within seconds and without the need for subjective interpretation of the data.

To establish ^19^F-paraGEST MRF, the Ln^3+^ chelator, α-CD-DTPA, was synthesized in a gram scale by combining two synthetic routes^51,52^ (Figures S1–S3), followed by the preparation of a 14-member library of Ln-α-CDs, modified with all available lanthanides.^48,50^ Next, ^19^F-paraGEST z-spectra were acquired with various saturation powers for solutions containing each Ln-α-CD and the fluorinated guest 3,5-difluorobenzylamine (**1**) with a 1:200 Ln-α-CD:**1** ratio, to ensure optimal data sets with minimal effects of back exchange. Fitting these data sets to Bloch–McConell eqs (Figure S4),^53^ a series of ^19^F-paraGEST parameters were extracted for each pair of Ln-α-CD:**1**, i.e., *T*_1_ [sec], *T*_2_ [sec], Δω [ppm], *f*_Ln_, and *k*_ex_ [sec^–1^] (Table S1), and were used for constructing the ^19^F-paraGEST MRF dictionary in the next stage. Interestingly, the exchange rate of **1** was found to be different between Ln-α-CDs, depending on the Ln^3+^ used, which might have been a result of the interactions of the amine group of **1** with the Ln^3+^ center inside the Ln-α-CD cavity.^48,50^ Different trends of exchange rates of labile ligands have been reported across the lanthanide series,^54–56^ with general observations showing increasing stability and inertness from light to heavy lanthanides, while other studies reveal deviations in water exchange behavior—most notably faster exchange in midlanthanides (Gd–Dy)—highlighting nontrivial findings that warrant further investigation of our system. This phenomenon can be utilized in the future for a better differentiation by the MRF algorithm to improve its performance and to make it more accurate in Ln^3+^ concentration determination.

A ^19^F-paraGEST MRF acquisition protocol was then designed (Figure 2a, Table S2), composed of 40 saturation points that are divided into three “blocks”: (block 1) points 1–15 include a set of acquisitions with a mild 1.75 μT saturation pulse applied at all 14 characteristic Δω′s (a defined Δω for each Ln-CD, determined from their z-spectra, Table S2) and an “off-resonance” saturation; (block 2) points 16–30 include the same acquisition scheme with a stronger 2.87 μT saturation pulse (corresponded to maximal ^19^F-paraGEST effects); (block 3) points 31–40 include a 2.87 μT saturation pulse with a shorter saturation time applied in the range of Δω = 1–11 ppm, at which multiple ^19^F-paraGEST effects are overlapping one another (Figure S5). The obtained data (Table S1) and the designed acquisition protocol (Figure 2a) served as the skeleton for simulating a preliminary dictionary of synthetic signal trajectories for each of the 14 lanthanides of interest in a broad and realistic range of concentrations (Table S3). At this stage, additional ^19^F-paraGEST MRF signal trajectories were computationally simulated for mixtures of two, three, and five Ln-α-CD hosts, which are often found in electronic waste samples,^57–59^ composed of all possible combinations of the 14 different lanthanides. The resulting simulated dictionary reflected 2471 combinations of lanthanides with various individual concentrations, yielding 7,026,509 entries (Table S3).

**Figure 2:**
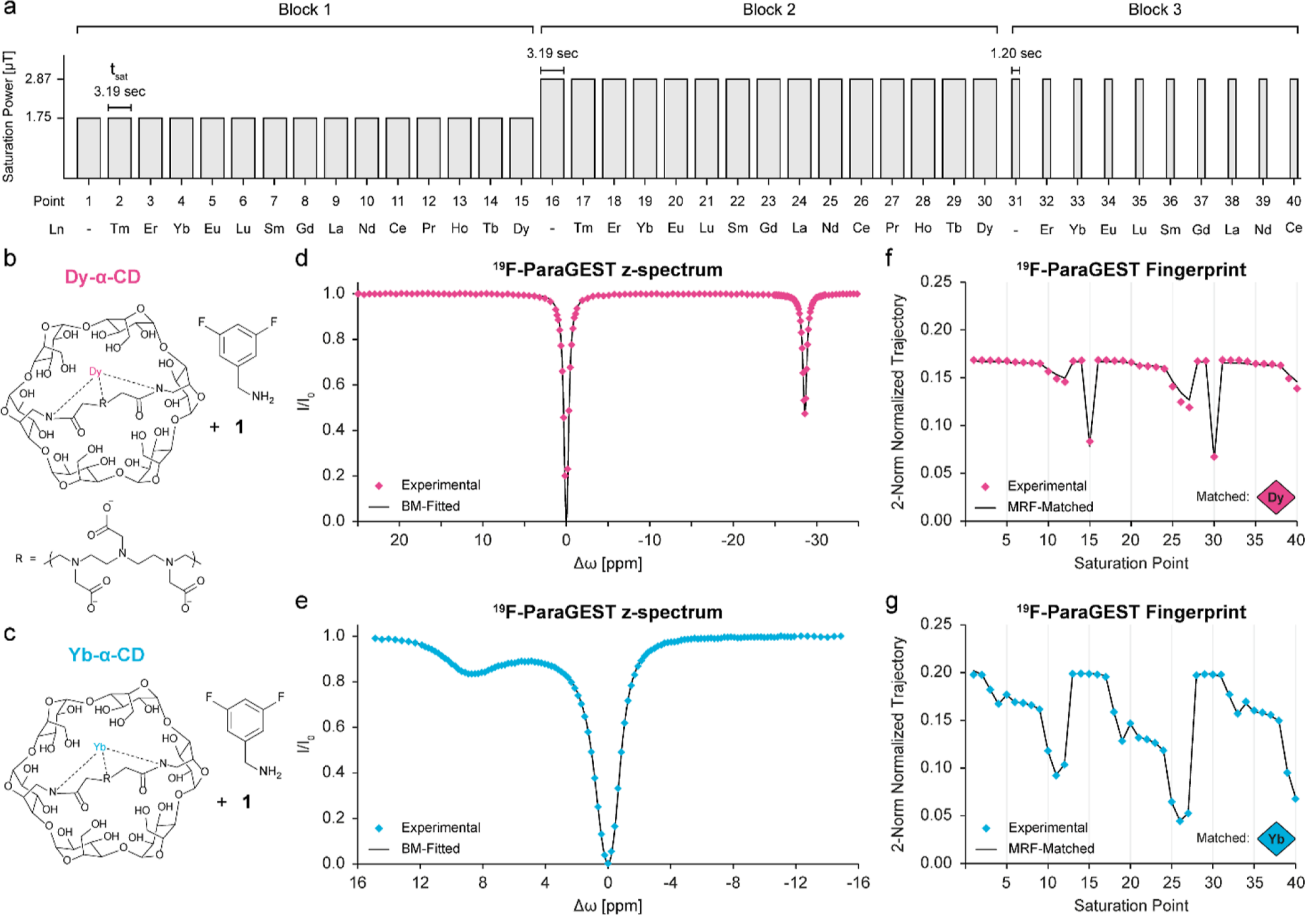
^19^F-paraGEST MRF characterization. (a) Scheme describing the designed ^19^F-paraGEST MRF acquisition protocol. Gray rectangles represent each saturation point (between 1 and 40), where the rectangle’s height represents the saturation power [μT], and its width represents the saturation time [sec]. All points were collected with a recovery time of 9.57 s between them; molecular structures for host–guest systems of (b) Dy-α-CD or (c) Yb-α-CD with guest **1** in a 1:200 ratio; experimental ^19^F-paraGEST z-spectra (points) and Bloch–McConnel fitting (line) for (d) Dy-α-CD:**1** and (e) Yb-α-CD:**1**; experimental (points) and matched (black line) ^19^F-paraGEST fingerprints for (f) Dy-α-CD:**1** and (g) Yb-α-CD:**1**; the lanthanide extracted from each matched fingerprint is represented as a colored diamond; all data was collected in an 11.7T NMR spectrometer.

The ability of ^19^F-paraGEST MRF to identify single lanthanides from their experimental fingerprints was then assessed for two samples containing molecular guest **1** and either Dy-α-CD (Figure 2b) or Yb-α-CD (Figure 2c). From the ^19^F-paraGEST z-spectra, the lanthanide content of each sample could be visually assigned based on the specific Δω of the dip of each effect (Table S1). Matching the ^19^F-paraGEST fingerprints to the dictionary entries (illustrated in Figure 1e–h) resulted in the correct lanthanide identification for each Ln-α-CD sample. The ^19^F-paraGEST effect of Dy-α-CD at −28.6 ppm (Figure 2d) is translated into local minima at points 15 and 20 in the MRF trajectory (Figure 2f), allowing the algorithm to identify the lanthanide (Figure 2f, diamond) correctly. Even when the ^19^F-paraGEST effect was broader and closer to 0 ppm, as for Yb-α-CD (Figure 2e), the simulated MRF trajectory (Figure 2g, black solid line) exhibits the same pattern as the experimental data (Figure 2g, diamonds), and the recognition algorithm was able to match between the two and identify the correct lanthanide in the solution.

Expanding this process for all of the lanthanides has revealed unique experimental fingerprints for each Ln-α-CD, followed by a pattern recognition matching process to identify each lanthanide (Figure 3a–l). Comparing the ^19^F-paraGEST MRF patterns, lanthanides with more significant pseudo contact shift (PCS, larger Δω), like Tb (Figure 3b) and Ho (Figure 3d), exhibit a sharp, clear trajectory. In comparison, lanthanides that produce paraGEST effects with smaller Δω′s have led to more complex patterns (for example, Lu and Sm, Figure 3f,g, respectively). All examined lanthanides, except Gd^3+^, were successfully identified by the algorithm with high dot product values (Table S4). For Gd-α-CD, a “shallow” ^19^F-paraGEST fingerprint, without clear peaks, was obtained (Figure 3k) that was wrongfully interpreted as a Ln-α-CD mixture of Gd and Pr (Figure 3k, diamonds). This sample assignment was not further optimized since Gd is rarely found as a single lanthanide in electronic, optical, and industrial waste. Nevertheless, future MRF studies could improve the assessment of Gd-containing samples by further exploiting and weighting the T_1_ and T_2_ relaxation rates expected by the significant paramagnetic properties of Gd^3+^ (e.g., using varied flip angles and repetition times).^60^

**Figure 3:**
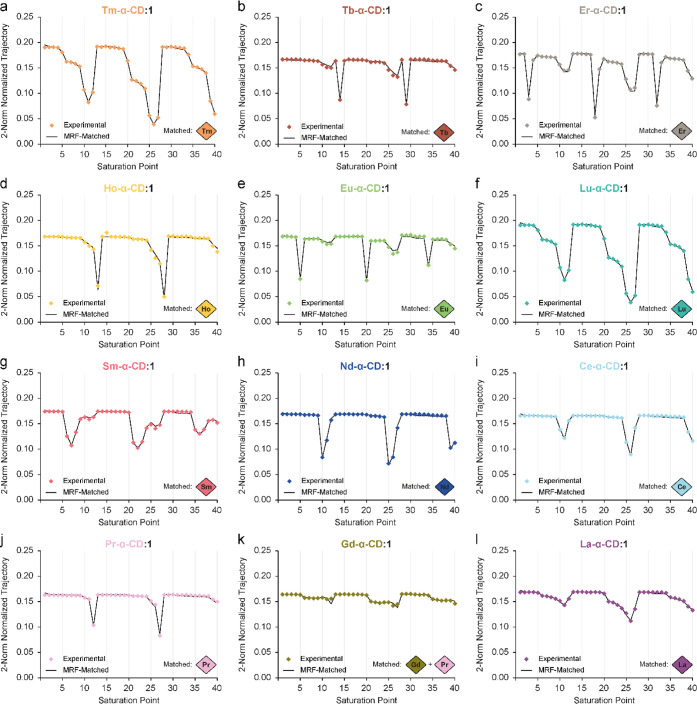
^19^F-paraGEST fingerprint analysis of samples containing single lanthanide. Experimental (points) and matched (line) ^19^F-paraGEST fingerprints for samples containing Ln-α-CD and guest 1 in a 1:200 ratio. Ln = (a) thulium; (b) terbium; (c) erbium; (d) holmium; (e) europium; (f) lutetium; (g) samarium; (h) neodymium; (i) cerium; (j) praseodymium; (k) gadolinium; and (l) lanthanum. Experimental data was collected in an 11.7T NMR spectrometer. The lanthanide extracted from each matched fingerprint is represented in a diamond.

Next, ^19^F-paraGEST MRF was applied to investigate mixtures of multiple lanthanides (Figures 4, S6–S8, Tables S5–S7). Twenty-two mixtures were fabricated and examined based on observed combinations in different wastes.^57,61^ Significantly, the same constructed dictionary (∼7 million entries, as described in Table S3) was blindly utilized to match the collected ^19^F-paraGEST MRF signals using computerized (human-intervention-free) pattern matching. First, samples containing combinations of two Ln-α-CDs (Figures 4a–c, S6, Table S5) were analyzed. A sample with a combination of Tb and Eu , which is often found in optical devices,^61^ was prepared, and its experimental fingerprint was identified correctly (Figure 4b). Furthermore, a mixture of Dy and Nd, which can be found in coal fly ash^62^ or permanent magnets,^63^ was prepared and successfully assigned (Figure 4c). Almost all the additional combinations tested (Figure S6) were successfully matched, presenting high dot products (Table S5). Noticeably, a mixture of La and Lu (Figure S6j) was correctly identified, even though the two selected lanthanides are diamagnetic, and their effects are only 0.6 ppm apart (Figure S5).

**Figure 4:**
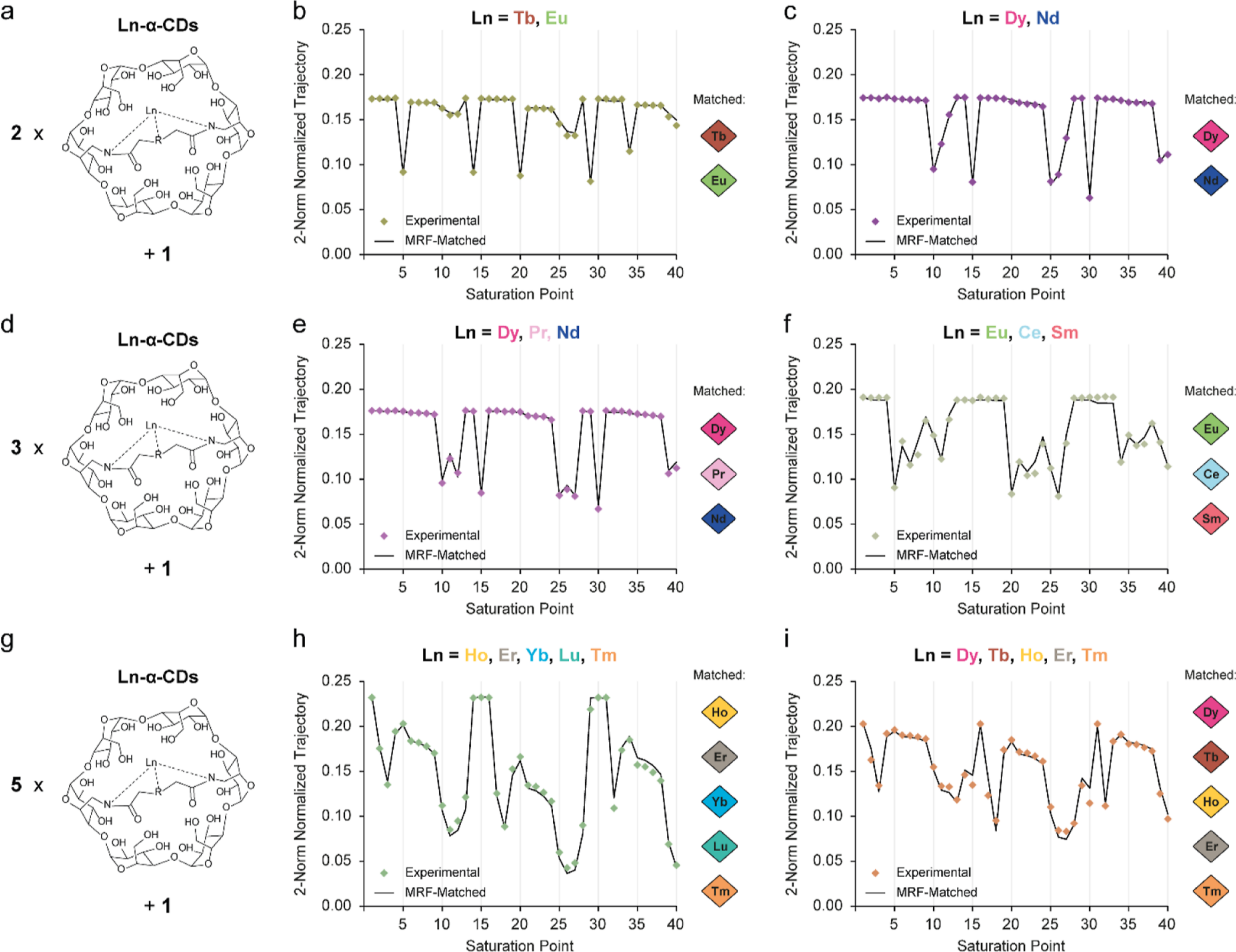
^19^F-paraGEST fingerprint analysis of samples containing lanthanide mixtures. (a) Scheme describing the content of samples containing two lanthanide mixtures; experimental (colored diamonds) and matched (black line) ^19^F-paraGEST fingerprints for samples containing Ln-α-CDs and guest **1** in a 1:1:200 ratio. Ln = (b) terbium and europium; (c) dysprosium and neodymium; (d) scheme describing the content of samples containing three lanthanide mixtures; experimental (colored diamonds) and matched (black line) ^19^F-paraGEST fingerprints for samples containing Ln-α-CDs and guest **1** in a 1:1:1:200 ratio. Ln = (e) dysprosium, praseodymium, and neodymium; (f) europium, cerium, and samarium; (g) scheme describing the content of samples containing five lanthanide mixtures; experimental (colored diamonds) and matched (black line) ^19^F-ParaGEST fingerprints for samples containing Ln-α-CDs and guest **1** in a 1:1:1:1:1:200 ratio. Ln = (h) holmium, erbium, ytterbium, lutetium, and thulium; and (i) dysprosium, terbium, holmium, erbium, and thulium. Experimental data was collected in an 11.7T NMR spectrometer. The lanthanides extracted from each matched fingerprint are represented in diamonds.

^19^F-ParaGEST MRF was then applied for combinations of three lanthanides (Figures 4d–f, S7, Table S6). Fingerprints for a mixture of Dy, Pr, and Nd, used in wind turbine generators,^61^ and a mixture of Eu, Ce, and Sm, which exists in waste cathode ray tubes,^64^ were recorded and correctly assigned (Figure 4e,f, respectively). An additional sample of Nd, Eu, and La (Figure S9b), used in surveillance equipment^61^ and solar transducers,^63^ was also successfully assigned. When the complexity of the samples was increased even more to mixtures of five different lanthanides, sample content was also identified by the ^19^F-paraGEST MRF method (Figures 4g–i, S8, Table S7). A mixture of Ho, Er, Yb, Lu, and Tm was assigned correctly (Figure 4h), showing the strength of ^19^F-paraGEST MRF for identifying lanthanides that cannot always be directly identified from the obtained ^19^F-paraGEST z-spectrum (Figure S9a). An additional combination of Dy, Tb, Ho, Er, and Tb was also successfully identified (Figures 4i and S9b). In other cases, like in a sample of Dy, Tb, Pr, Nd, and Sm (Figure S9), which can be found in hybrid vehicles,^65^ four out of the five lanthanides were identified, as Pr was recognized as Lu. This is because the experimental fingerprints have become more crowded in a solution with such a combination, making the matching process more prone to errors, a challenge that might be overcome with a different ^19^F-paraGEST MRF acquisition protocol. Nevertheless, although ^19^F-ParaGEST MRF misassigned one Ln^3+^ (out of 5) in one of the examined 5-Ln^3+^ mixtures, it shows high accuracy when applied to identify lanthanides in industrial-relevant mixtures (Figure 4). To evaluate the accuracy of the simulations, we performed a fidelity estimation of the data model agreement across the entire experimental data and its matched simulated counterparts, as performed in similar computational tasks.^66^ The resulting coefficient of determination for the examined cases was *R* ^2^ = 0.9996 ± 0.0005 (mean ± standard deviation for the coefficient of determination calculated across all studied cases), reflecting an excellent fidelity.

Finally, realizing that lanthanide-containing permanent magnets, which are essential components in hard disk drives (HDDs), are a subject for lanthanide mining,^57,67–71^ we applied ^19^F-paraGEST MRF to identify the lanthanide substance in a HDD. Two permanent magnets were first isolated from a Western Digital HDD, demagnetized, and crushed into small pieces (Figures 5a and S10a–d). Then, the lanthanide content was extracted by adapting an acid-leaching process (for a detailed method, see Supporting Information),^63^ resulting in an “unknown” mixture (Figures 5b and S10e–g), followed by the addition of the α-CD-DTPA chelator (Figure S1g) to obtain a mixture of Ln-α-CDs of the HDD’s leached lanthanides (Figure 5c). Guest **1** was added to that mixture, and the ^19^F-paraGEST MRF acquisition protocol (Figure 2a) was applied to obtain a characteristic signal evolution pattern that can be analyzed (Figure 5d) to reveal the lanthanide composition of the HDD’s permanent magnets (Figure 5e). The experimental ^19^F-ParaGEST MRF signal trajectory of the “unknown” solution showed the highest correlation to a single entry of the entire dictionary (Figure 5f).

**Figure 5:**
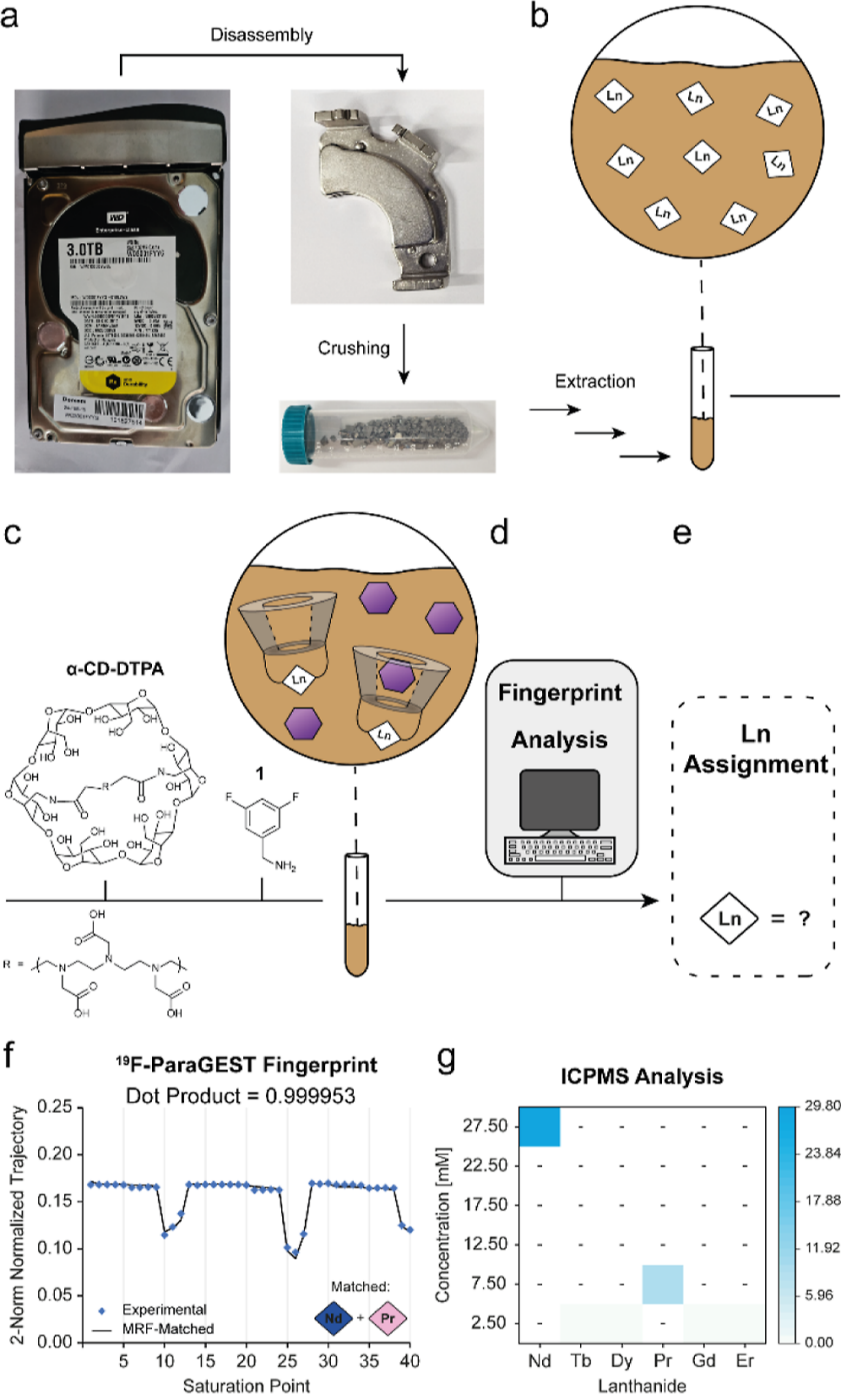
Lanthanide detection in electronic waste. Scheme describing the detection process for a hard disk drive (HDD): (a) permanent NdFeB magnet is separated from the HDD, demagnetized, and crushed into small pieces; (b) a mixture of lanthanide ions is leached from the magnet pieces using acid; (c) α-CD-DTPA chelator and **1** are added to the mixture, where the α-CD-DTPA complexed with the free lanthanides, resulting in multiple host–guest systems; (d) the experimental ^19^F-paraGEST fingerprint is collected and matched to the dictionary entries; and (e) the identity of the leached lanthanides is revealed by the algorithm. (f) Experimental (colored diamonds) and matched (black line) ^19^F-paraGEST fingerprint for the HDD extraction (11.7T) and the identified lanthanides and (g) results of lanthanide composition ICPMS analysis for the HDD extraction. The color bar in the right-hand *y*-axis represents concentrations.

The matched fingerprint was simulated for two lanthanides, revealing the HDD’s permanent magnets consisting mainly of Nd^3+^ and Pr^3+^. The ^19^F-ParaGEST z-spectrum was recorded for the same sample to validate the result further, illustrating the effects of the corresponding lanthanides (Figure S11). It is important to mention here that adding an excess of α-CD-DTPA will not affect the obtained results since a host without complexed Ln^3+^ will not affect the paraGEST effect. In contrast, α-CD-DTPA at concentrations lower than that of the Ln^3+^ content in the solution can lead to underestimation of the ion of interest, since noncomplexed lanthanides do not generate paraGEST effects. To examine the reliability of the ^19^F-paraGEST MRF results (Figure 5f), the lanthanide content of the “unknown” solution was analyzed using inductively coupled plasma mass spectrometry (ICP-MS, Figure 5g and Table S8). As for the MRF data, ICP-MS identified Pr^3+^ and Nd^3+^ as the two major lanthanides of the permanent magnets, with detected traces of Tb^3+^, Dy^3+^, Gd^3+^, and Er^3+^, which are presented in concentrations that are 2 orders of magnitude lower than Nd^3+^ and an order of magnitude lower than Pr^3+^, emphasizing the ultimate sensitivity of ICP-MS. Interestingly, although the permanent magnets used in HDDs are mainly NdFeB magnets,^71^ Pr^3+^ is often used to create Nd–Pr alloys in NdFeB magnets,^67,72^ in correlation with our ^19^F-paraGEST MRF findings. Overall, we have shown here that the ^19^F-paraGEST MRF can accurately identify the significant Ln^3+^ content expected in electronic waste.

## Conclusions

To conclude, we designed, constructed, and tested a new paradigm titled ^19^F-paraGEST MRF for the detection of free lanthanides in solutions using NMR (Figure 1). Adapting and expanding CEST-MRF principles to ^19^F-paraGEST of supramolecular Ln-α-CD:**1** pairs, the characteristic ^19^F-paraGEST parameters for each pair were used to generate a synthetic “dictionary” consisting of 7,026,509 simulated entries of signal trajectories for all possible host–guest pairs, and for combinations of two, three, or five different Ln-α-CDs with **1**, in various lanthanide concentrations. The combination of a relatively short NMR acquisition protocol and accurate data simulation circumvented the need for a laborious acquisition of experimental reference data sets of 2471 lanthanide combinations (in ∼7 M different concentration variants), providing a high-throughput approach for automatic identification of lanthanides. Notably, the ^19^F-paraGEST MRF acquisition time was shorter than required for a complete z-spectrum acquisition, and the automatic pattern recognition for all examined samples took merely 18.46 s when implemented on a standard laptop.

The performances of ^19^F-paraGEST MRF identifying Ln^3+^ ions in a solution were blindly tested on experimental solutions prepared with each of the Ln-α-CDs (Figures 2f,g and 3) and their mixtures (Figures 4 and S6–S8), where the approach showed a high identification degree by successfully matching the experimental fingerprints to simulated entries in the dictionary. The potential of the ^19^F-paraGEST MRF for detecting lanthanides in a recyclable material was tested on permanent magnets from an HDD (Figure 5), successfully detecting Nd^3+^ and Pr^3+^, expected in Nd–Pr alloys of such magnets. Overall, the ^19^F-paraGEST MRF shows unique abilities for detecting lanthanides in different combinations and should be further applied in scenarios involving sustainable resource management and rare-earth element recovery. While ICP-MS remains the gold standard for lanthanide analysis due to its high sensitivity, accuracy, and relatively low cost, it is inherently sample-destructive. In contrast, our ^19^F-paraGEST MRF approach is nondestructive, allows for sample recovery, and may benefit from the growing availability of compact, cost-effective benchtop NMR systems, positioning it as a promising complementary tool for lanthanide detection in settings where sample preservation or on-site analysis is valuable. Demonstrated here for detecting lanthanides, the GEST-MRF approach, which leverages dynamic supramolecular interactions with MRF, paves the way for a broad spectrum of applications in molecular sensing, enabling the detection of diverse analytes and expanding the frontiers of MR-based methodologies.

## Supplementary Material





## Notes

Chemicals used, synthetic procedures, sample preparations, NMR studies, MRF dictionary generation, MRF matching, ICP-MS, and method applied for lanthanide extraction from hard disc (PDF)
^19^F-ParaGEST MRF experimental data sets (ZIP)
