# Supplementary material for: Harnessing Dynamic Supramolecular Interactions for Lanthanide Detection via Computational Pattern Recognition of Magnetic Resonance Fingerprints
Source: J Am Chem Soc. 2025 May 19;147(22):18972–81. doi: 10.1021/jacs.5c03583 (PMC12147137; doi:10.1021/jacs.5c03583)
Supplement: Supplementary file 1 [file ja5c03583_si_001.pdf]

Supporting Information

Harnessing Dynamic Supramolecular Interactions for Lanthanide Detection via Computational Pattern Recognition of Magnetic Resonance Fingerprints

Elad Goren,[1] Balamurugan Subramani,[1] Liat Avram,[2] Alla H. Falkovich,[2] Or Perlman,[3,4*] Amnon Bar-Shir[1*]

[1] Department of Molecular Chemistry and Materials Science, Weizmann Institute of Science, Rehovot, 7610001, Israel; [2] Department of Chemical Research Support, Weizmann Institute of Science, Rehovot, 7610001, Israel; [3] Department of Biomedical Engineering, Tel Aviv University, Tel Aviv, 6997801, Israel; [4] Sagol School of Neuroscience, Tel Aviv University, Tel Aviv, 6997801, Israel

*Corresponding Authors: amnon.barshir@weizmann.ac.il and orperlman@tauex.tau.ac.il

Supporting Methods

Abbreviations

CD – Cyclodextrin, NaH – Sodium hydride, DTPAA - Diethylenetriaminepentaacetic acid dianhydride, DMSO – Dimethyl Sulfoxide, NaN_3 - Sodium azide, DIBAL-H - Diisobutylaluminum hydride, DMF – dimethylformamide, N_2 – Nitrogen, RT – room temperature, TLC – thin layer chromatography, HCl - hydrochloric acid, H_2O – water, DCM – dichloromethane, TEA – Triethylamine, THF – tetrahydrofuran, Pd/C – palladium on activated carbon, H_2 – hydrogen, $LnCl_3 \cdot XH_2O$ – Lanthanide Chloride hydrate (X = 6/7, depends on the lanthanide), ddW - doubly de-ionized water.

Chemicals

α-cyclodextrin (α-CD), Benzyl bromide, Neodymium (III) chloride hydrate, Samarium (III) chloride hydrate, Lutetium (III) chloride hexahydrate and 3,5-Difluorobenzylamine (**1**) were purchased from Alfa Aesar. NaH, DTPAA and DMSO anhydrous were purchased from Sigma Aldrich. Mesylchloride, NaN_3, Cerium (III) chloride heptahydrate was purchased from Acros Organics. DIBAL-H was purchased from Thermo Scientific. Praseodymium (III) chloride heptahydrate, Europium (III) chloride hexahydrate, Terbium (III) chloride hydrate, Dysprosium (III) chloride anhydrous, Holmium (III) chloride hexahydrate, Erbium (III) chloride hydrate, Thulium (III) chloride hydrate, and Ytterbium (III) chloride hexahydrate were purchased from Strem Chemicals INC. Lanthanum (III) chloride heptahydrate was purchased from Fisher Chemical.

Perbenzylated-α-CD Synthesis

NaH (14.8 g, 60 eq) was added in three portions to a stirred solution of α-CD (10 g, 10.28 mmol) in anhydrous DMF (200 mL), under N_2 at 0°C. Benzyl bromide (50 mL, 411.16 mmol) was then added dropwise over 30 min. The reaction was mixed overnight, warmed to RT, and mixed for additional two days. Following verification of conversion with TLC, the reaction was carefully hydrolyzed with brine (50 mL) and diluted with brine (200 mL) and extracted with ethyl acetate (4×200 mL). The combined organic layers were washed with brine and H_2O alternately (six times total), dried using $MgSO_4$, filtered, and concentrated. After purification by silica gel chromatography (0-40% ethyl acetate in hexane) and drying under high vacuum,

perbenzylated-α-cyclodextrin was obtained as a brownish wafer (21.39 g, 80.18 %); HRMS: $C_{162}H_{168}O_{30}$ calcd: m/z 2593.16, found: 2616.15 $[M+Na]^+$, Δ 1.0 ppm.

$6^A,6^D$-Dihydroxy-α-cyclodextrin (Dihydroxy-α-CD) Synthesis

DIBAL-H (106.64 mL 1.2M in toluene, 130 mmol) was added slowly to dried perbenzylated-α-CD (11.07 gr, 4.26 mmol), and the mixture was stirred at 50°C under N_2. The reaction was carefully monitored by TLC (30% ethyl acetate in hexane), and the mixture was hydrolyzed after five hours by dropwise addition of HCl 1M at 0°C. Crude solution was extracted twice with ethyl acetate and the combined organic phase was washed twice with brine and dried with $MgSO_4$. After purification by silica gel chromatography (0-24% ethyl acetate in hexane), the compound (7.96 gr, 72.29 %) was obtained as a yellowish wafer; HRMS: $C_{148}H_{156}O_{30}$ calcd: m/z 2413.07, found: 2436.06 $[M+Na]^+$, Δ -0.2 ppm.

$6^A,6^D$-Dichloro-α-cyclodextrin (Dichloro-α-CD) Synthesis

To a solution of dihydroxy-α-CD (7.96 g, 3.30 mmol) in anhydrous DCM (160 mL) under N_2, TEA was added (2.76 mL, 19.78 mmol). Then, mesylchloride (2.04 mL, 26.37 mmol) was added slowly in two portions and the mixture was stirred at RT for four hours. DCM was then removed under vacuum; the solution was dried under high vacuum and anhydrous DMF (40 mL) was added to the residue. The mixture was stirred at 100 °C overnight under N_2, followed by dilution with ethyl acetate and brine (200 mL each). The organic phase was washed four times with brine and water alternately, dried with $MgSO_4$, filtered, and evaporated. Purification of the product by silica gel chromatography (0-50% ethyl acetate in hexane) gave the product as a yellow-orange wafer (6.63 gr, 81.94%); MS: $C_{148}H_{154}O_{25}Cl_2$ calcd: m/z 2449.00, found: 2472.01 $[M+Na]^+$.

$6^A,6^D$-Diazido-α-cyclodextrin (Diazido-α-CD) Synthesis

To a solution of dichloro-α-cyclodextrin (6.63 gr, 2.70 mmol) in anhydrous DMF (37 mL) under N_2 was added NaN_3 (1.41 g, 21.63 mmol). The reaction mixture was stirred at 80 °C in an oil bath overnight, then cooled down to RT, evaporated and dried under high vacuum. The residue was dissolved in ethyl acetate (100 mL) and washed with water (3×100 mL). The aqueous layer was extracted with ethyl acetate (3×100 mL). The combined organic layers were dried over $MgSO_4$, filtered, and concentrated. Silica gel chromatography of the residue (0-30% ethyl acetate in hexane) gave the diazido-α-CD (5.86 g, 88.05 %) as a yellowish wafer; MS: $C_{148}H_{154}O_{28}N_6$ calcd: m/z 2463.08, found: 2486.07 $[M+Na]^+$.

$6^A,6^D$-Diamino-α-cyclodextrin (Diamino-α-CD) Synthesis

Diazido-α-CD (1.04 gr, 420 mmol) was dissolved in a mixture of H_2O (20 mL) and THF (60 mL) in a Fisher-Porter tube and then TFA (250 µL) and Pd/C 10% (505.60 mg) were added. The reaction mixture was stirred under an H_2 atmosphere (2 bar) overnight, and under 3.5 bar pressure for an additional night. After TLC (30% ethyl acetate in hexane) showed full consumption of the starting material, the solution was filtered through a celite pad and washed with H_2O. The filtrate was further filtered through a filter paper and concentrated under vacuum. The remaining aqueous solution was lyophilized, then dissolved in a minimum amount of DMF and

re-precipitated from cold acetone to give diamino-α-CD (1.00 gr) as an amorphous powder; HRMS: $C_{36}H_{62}O_{28}N_2$ calcd: m/z 970.35, found: 971.36 $[M+H]^+$, Δ 0.9 ppm.

6^A,6^D-Diethylenetriaminepentaacetic-α-cyclodextrin (α-CD-DTPA) Synthesis

Under N_2, a solution of diamino-α-CD (100 mg, 95.80/82.92 μmol), DTPAA (34.23/29.63 mg, 95.80/82.92 μmol) and TEA (one drop) was stirred in anhydrous DMSO (2 mL) overnight. The solution was added dropwise into cold acetone (60 mL) while stirring, and the resultant white powder was collected after centrifugation (4˚C, 3000 rpm, 15 min). The crude solid was dissolved in a minimal volume of water and purified through reversed-phase liquid chromatography. α-CD-DTPA was isolated as a white powder upon the removal of solvent (13.74 mg, 21.6% / 18.77 mg, 22.78%); HRMS: $C_{50}H_{81}O_{36}N_5$ calcd: m/z 1327.4713, found: 1328.4739 $[M+H]^+$, Δ -2.0 ppm, 1350.4534 $[M+Na]^+$, Δ -1.9 ppm, 1366.4233 $[M+K]^+$, Δ -4.8 ppm.

Lanthanide-modified-α-cyclodextrin (Ln-α-CD) Synthesis

For each lanthanide, a solution of α-CD-DTPA (one equivalent) and $LnCl_3•XH_2O$ (one equivalent) in 15 mL ddW was refluxed under stirring for one hour. Ln-α-CD (90-100%) was isolated as a white powder upon the removal of solvent; HRMS: For Dy-α-CD - $C_{50}H_{79}O_{36}N_5Dy$ calcd: m/z 1489.3796, found 1489.3787 $[M+H]^+$, Δ -0.6 ppm.

Reversed-Phase High-Pressure Liquid Chromatography (RP-HPLC)

Analytical RP-HPLC analysis was performed using an Agilent Technologies 1260 Infinity quaternary pump LC system equipped with a diode-array detector through a C18 column. Preparative RP-HPLC was carried out using an Agilent 218 purification system equipped with an auto-sampler, a C18 column, a UV-Vis dual-wavelength detector, and a 440-LC fraction collector, operating under OpenLab ChemStation software. Elution phases were composed of 0.1% TFA in ddW (eluent A) and 90% acetonitrile, and 0.1% TFA in ddW (eluent B).

High-Resolution Electrospray Ionization Mass Spectrometry (ESI-Q-Tof-MS)

Analyses were carried out on a Waters Xevo G2-XS QTof Mass Spectrometer (Manchester, UK) with an electrospray ionization (ESI) source operating in the positive mode. Solutions were directly infused at a flow rate of 10 μL/min. All spectra were acquired in a mass range of 50 – 2000 or 350-5000 m/z depending on the required mass. Mass errors of the analyzed spectra were no larger than 5.0 ppm. The analyses were performed using a capillary voltage of 2.2 – 3.00 kV, a cone gas flow of 50 L/hr, a source temperature set at 120˚C, and a cone voltage of 20V. The desolvation temperature was set at 250 - 280˚C, and the desolvation gas (N_2) flow rate was set to 600 L/hr. All measurements were done using Leucine-Enkephalin (200μg/uL, acetonitrile : H_2O containing 0.1% formic acid (1:1, v/v)) as a lock spray reference at a flow rate of 10uL/min to ensure mass accuracy and to follow resolution mode. Data acquisition and recording were made by Waters MassLynx v4.2 software.

High-Resolution Nuclear Magnetic Resonance (NMR)

a. <u>*Sample Preparation:*</u> Unless stated otherwise, all NMR experiments were performed on solutions containing Ln-α-CDs and guest **1** dissolved in a mixture of 10% D_2O and 90% H_2O. Solutions were prepared in a 1:200 host-guest molar ratio with final concentrations of 86 μM and 17.2 mM, respectively. For experiments with multiple Ln-CDs, each Ln-CD was prepared in an 86 μM concentration (i.e., 1:1:200 host-guest ratio for two lanthanides, 1:1:1:200 for three lanthanides and 1:1:1:1:1:200 for five lanthanides). All solutions were filtered using a MILLEX®-GV 0.22 μm PVDF filter unit before use, and the pH was measured and found to be pH=10 for all studied samples.

Unknown samples were prepared by mixing lanthanide extraction (C_f = 355.37 μM for Nd and C_f = 105.80 μM for Pr) with D_2O and H_2O and titrating the solution with TEA and HCl to a final pH of 6.46. The solution was filtrated to remove orange FeOH precipitants and then refluxed with α-CD-DTPA (C_f = 375.37 μM) for 1h. After cooling, guest **1** was added (C_f = 17.2 mM) and the solution was stirred overnight at room temperature. Following that, the solution was filtered again to remove excess guest precipitations and titrated again using TEA to a final pH of 9.38.

b. <u>*Data Acquisition:*</u> All NMR experiments were performed on an 11.75 T AVANCE III-HD NMR spectrometer (Bruker, Germany) with the sample temperature stabilized at 298K unless stated otherwise. *1D ^{1}H-NMR* spectra [500.08 MHz] were acquired for all samples before the ^{19}F-NMR experiments. *1D ^{19}F-NMR* spectra [470.54 MHz] were obtained for all host-guest samples, followed by longitudinal (T_1) and transverse (T_2) relaxation time evaluations. For structure assignment, *^{13}C[^{1}H]-NMR* spectra [125.74MHz] were measured with a spectral width of 250 ppm, acquired with 64K points, and a recycle delay of 5 seconds.

c. *Relaxation Times*: Prior to all ^{19}F-paraGEST experiments, both ^{19}F-inversion recovery (IR, for evaluating T_1) and ^{19}F-Car–Purcell–Meiboom–Gill (CPMG, for evaluating T_2) experiments were performed.

d. *^{19}F-ParaGEST* experiments: Following a repetition time of 9.57 sec, a presaturation continuous wave (CW) radiofrequency (RF) pulse, with a duration of 3.19 sec, was applied before the 90° radiofrequency pulse. The saturation pulse strength (B_1) was defined for each experiment as mentioned in the figure. To acquire the full z-spectrum, the frequency of the presaturation pulse was swept from Δω = (+n) ppm to Δω = (-n) ppm offset relative to the resonance frequency of the free guest (n = 35 for Dy; n = 25 for Tb; n = 23 for Tm; n = 16 for Ho; n = 15 for Er and Yb; n = 8 for Eu; n = 5 for Lu, Sm, Nd, Ce, Gd and La, and n = 3 for Pr). In addition, a ^{19}F-NMR spectrum where the RF presaturation pulse was applied at ω = -0.21 ppm (Δω = 110.52 ppm), where a saturation transfer effect was not expected, was acquired as a reference spectrum.

e. *^{19}F-ParaGEST MRF* experiments: Following a repetition time of 9.57 sec, a presaturation CW RF pulse, with a duration of 3.19 sec (points 1-30)/1.20 sec (points 31-40), was applied before the 90° RF pulse. B_1 was set to 1.75 μT (points 1-15) or 2.87 μT (points 16-40). The frequency of the presaturation pulse was swept between different Δω offsets relative to the resonance frequency of the free guest, as mentioned in Table 1. In addition, points 1, 16 and 31 include ^{19}F-NMR spectra in which the RF presaturation pulse was applied at Δω = 300 ppm (where a saturation transfer effect was not expected) and were used as reference spectra.

f. *Data Processing:* The z-spectra and fingerprint were blueprinted for each experiment by plotting the normalized intensity (I/I_0) of the ^{19}F-free guest signal at each frequency offset ($\Delta\omega$ relative to the frequency of the free guest), as a function of this applied presaturation pulse offset.

g. *Bloch-McConnell data fitting:* To estimate the ^{19}F-ParaGEST parameters (Table S1), the z-spectra of multi B_1 ^{19}F-ParaGEST experiments were fit using the Bloch–McConnell equations.[1] Analytical simulations were performed on the z-spectra using custom-written scripts in MATLAB version 23.2.0.2668659 (The MathWorks, Natick, MA) and the following parameters were extracted: $\Delta\omega$, k_{ex}, f_B, R_1 (bound guest), and R_2 (bound guest) . The code for data fitting can be found at http://www.cest-sources.org/doku.php?id=start and follows the publication by Zaiss and Bachert.[2, 3]

MRF Dictionary Generation

Simulated signal trajectories were generated using a flexible multi-proton-pool Bloch–McConnell equations numerical solver, implemented in C++ with a Python front-end and parallelization capabilities based on the pulseq standard.[4] Simulated trajectories for 14 different single lanthanides were generated using the guest exchange rate, T_1, T_2, and chemical shift offsets obtained from multi-B_1 z-spectra fitting (Table S1), yet with 750 different concentrations for each lanthanide, yielding a total of 10,500 different dictionary entries (Table S3).

Next, the dictionary was expanded to include all 2,471 possible combinations of one, two, three, and five Ln's, using a 6-pool Bloch-McConnell simulation with the concentration ranges described in Table S3, resulting in a total of 7,026,509 different dictionary entries. The dictionary can be reproduced using the open-source code available in https://github.com/momentum-laboratory/molecular-mrf[4] and the parameters described in Tables S1-S3.

Fingerprint Matching (Lanthanide Assignment)

The pattern matching methodology for assigning an experimentally measured NMR trajectory to a specific dictionary entry utilized the vector dot product (DP) after 2-norm normalization metric:[5, 6] DP $(\mathbf{e}, \mathbf{d}) = \langle \mathbf{e}, \mathbf{d} \rangle / (\|\mathbf{e}\| \cdot \|\mathbf{d}\|)$, where **e** denotes an experimental signal trajectory, and **d** denotes the dictionary entry vector. The computation was performed by matching all 43 experimental NMR signals against all 7,026,509 dictionary entries at once and took 18.46 sec using a laptop equipped with 16 CPU threads and 32G RAM. All experimentally measured MRF signal trajectories are provided in the supporting file dataTomatch.mat. The dot-product computation can be reproduced using the open-source code available in https://github.com/momentum-laboratory/molecular-mrf.[4]

Inductively Coupled Plasma Mass Spectrometry (ICPMS)

The sample solutions were analyzed for element concentrations using inductively coupled plasma mass spectrometry (ICP-MS, Agilent 7700). Drift corrections were carried out using indium (^{115}In) as an internal standard and by repeatedly analyzing a calibration solution of 50 µg L^{-1} concentration as a drift monitor throughout the analysis. Element concentrations were back calculated from the linear regression using

calibration curves plotted for each element within 1 – 1000 μg L^{-1} range. Standard solutions of 1, 5, 10, 50, 100, 500 and 1000 μg L^{-1} were prepared by consequent dilutions of IV-Stock-26 (Inorganic Ventures, US). The following masses were measured ^{139}La, ^{140}Ce, ^{146}Nd, ^{153}Eu, ^{159}Tb, ^{163}Dy, ^{89}Y, ^{141}Pr, ^{147}Sm, ^{157}Gd, ^{165}Ho, ^{166}Er, ^{169}Tm, ^{172}Yb and ^{175}Lu.

Lanthanide Extraction

Western Digital Re WD3001FYYG 3TB Hard disk drive (HDD, Fig S15a) was disassembled and the NdFeB magnets, sitting in a metal frame, were separated (Fig. S15b). Demagnetization was then conducted by heating the parts at 300°C for 1h.[7] After cooling, the magnets were taken out from the frame (Fig. S15c) and crushed with a hammer into small pieces (Fig. S15d). Extraction was performed based on a published procedure.[8] 1.04 gr of crushed magnet pieces were dissolved in 10 mL of 1M HNO_3 by stirring the mixture for 2h at room temperature. During this time, the liquid changed its color from colorless to dark yellow and finally to dark green (Fig. S15e-f). The solution was filtered from the remaining solids and was titrated with 1M NaOH until pH 2-3. FeOH precipitants were observed as black solids and were removed using filtration.

Supporting Figures

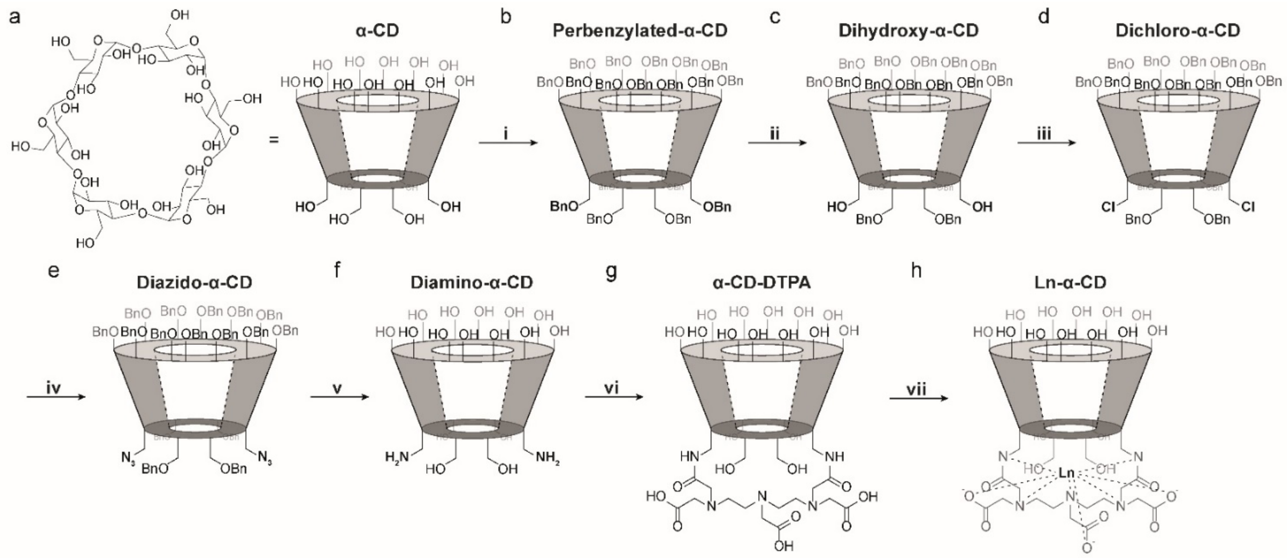

Figure S1. Synthesis of lanthanide-modified α-CDs (Ln-α-CD). (a) Molecular structure and schematic representation for α-CD; (b) Synthesis of perbenzylated-α-CD, i = Benzylbromide, NaH, anhydrous DMF, mixed overnight at room temperature; (c) Synthesis of dihydroxy-α-CD, ii = DIBAL, Toluene, mixed 5 hours at 50°C; (d) Synthesis of dichloro-α-CD, iii = 1. MsCl, TEA, anhydrous DCM, mixed at room temperature for 4 hours, 2. Anhydrous DMF, mixed overnight at 100°C; (e) Synthesis of diazido-α-CD, iv = NaN_3, anhydrous DMF, mixed overnight at 80°C; °C; (f) Synthesis of diamino-α-CD, v = 10% Pd/C, TEA, H2O, mixed under H_2 atmosphere for 2 days at room temperature; (g) Synthesis of α-CD-DTPA, vi. DTPAA, TEA, anhydrous DMSO, mixed for 16 hours at room temperature; (h) Synthesis of Ln-α-CD, vii. reflux in aqueous Lanthanide chloride ($LnCl_3$) solution for 1 hour, -Hydrochloric acid (HCl).

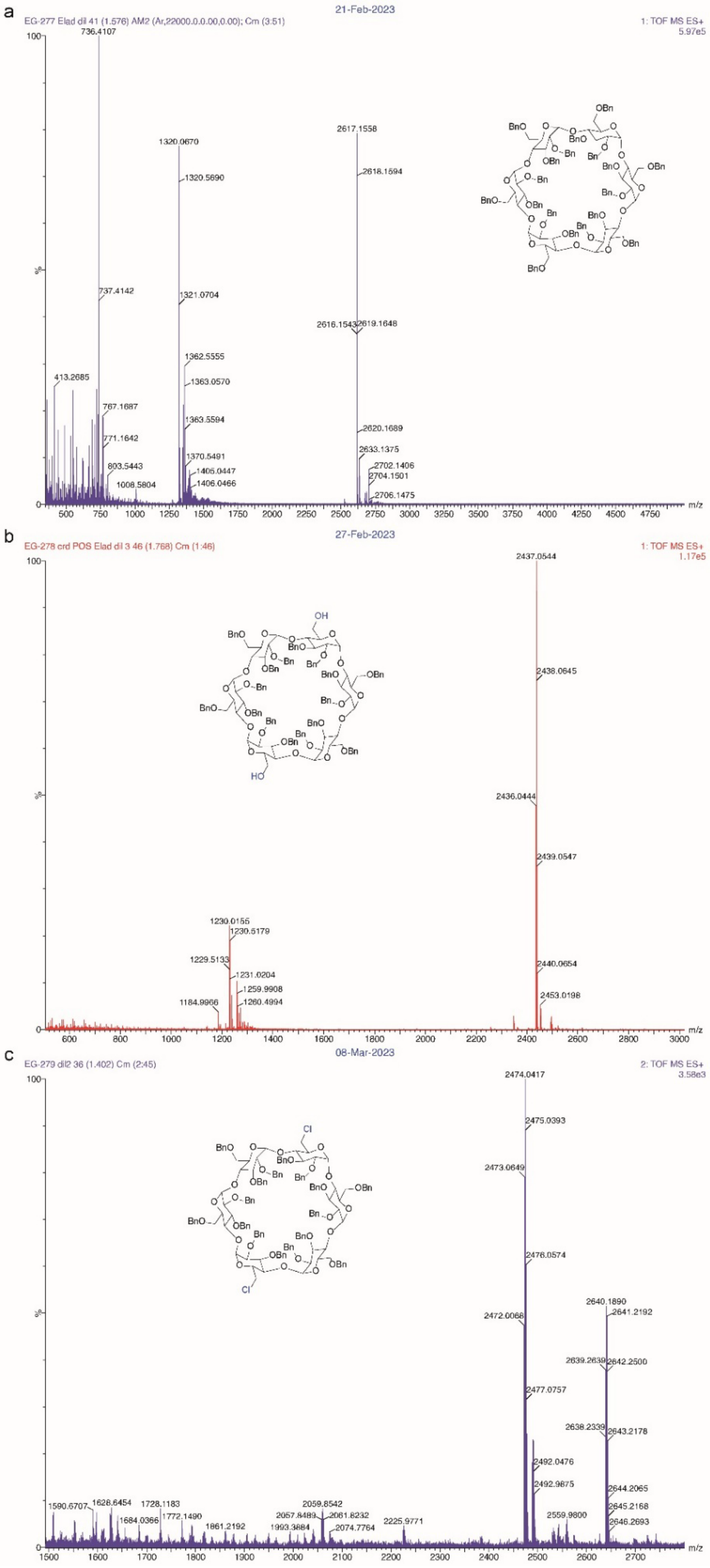

Figure S2. Full range MS spectra for (**a**) perbenzylated-α-CD, (**b**) dihydroxy-α-CD, and (**c**) dichloro-α-CD.

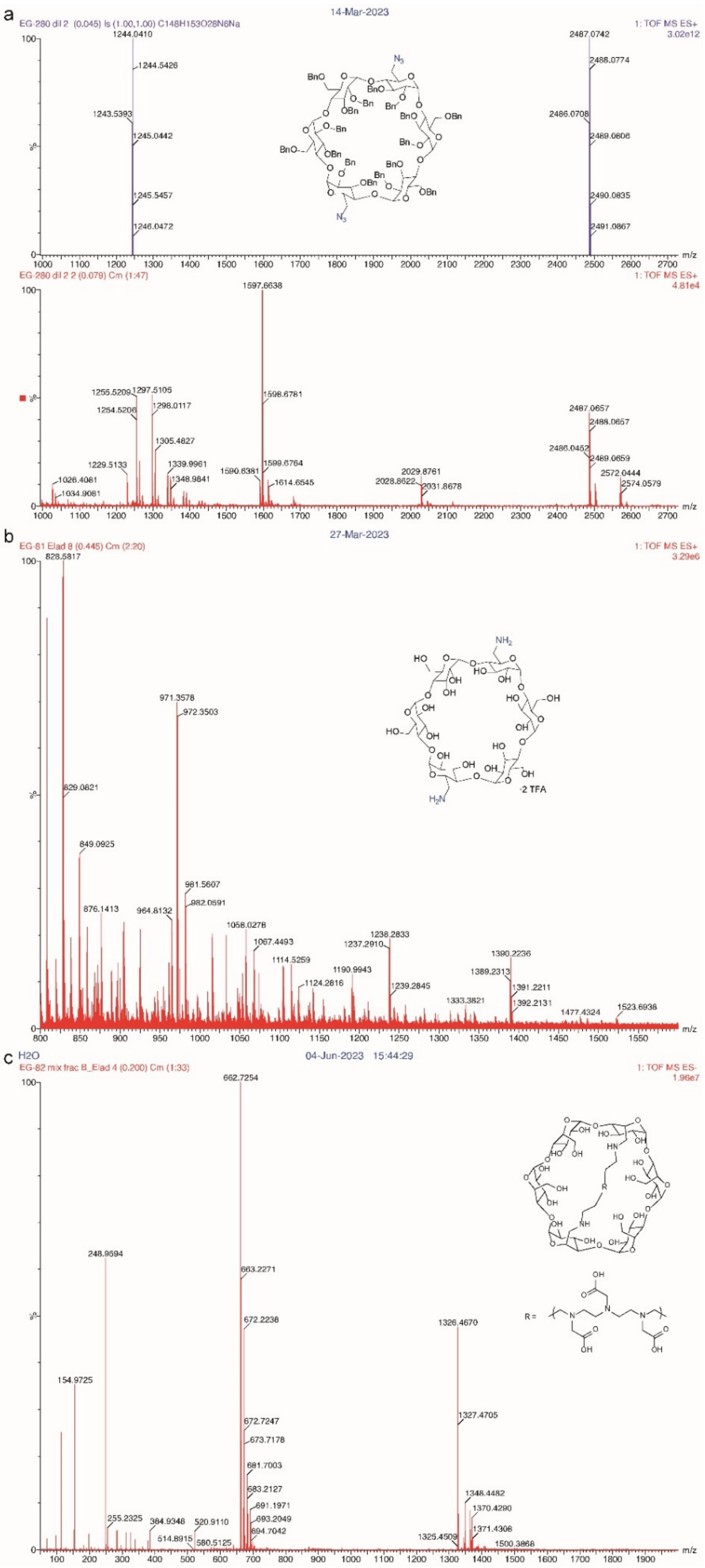

Figure S3. (**a**) Isotopic patterns (top) and experimental mass distribution (bottom) for diazido-α-CD; (**b**) full range MS spectrum for diamino-α-CD; (**c**) full range MS spectrum for α-CD-DTPA.

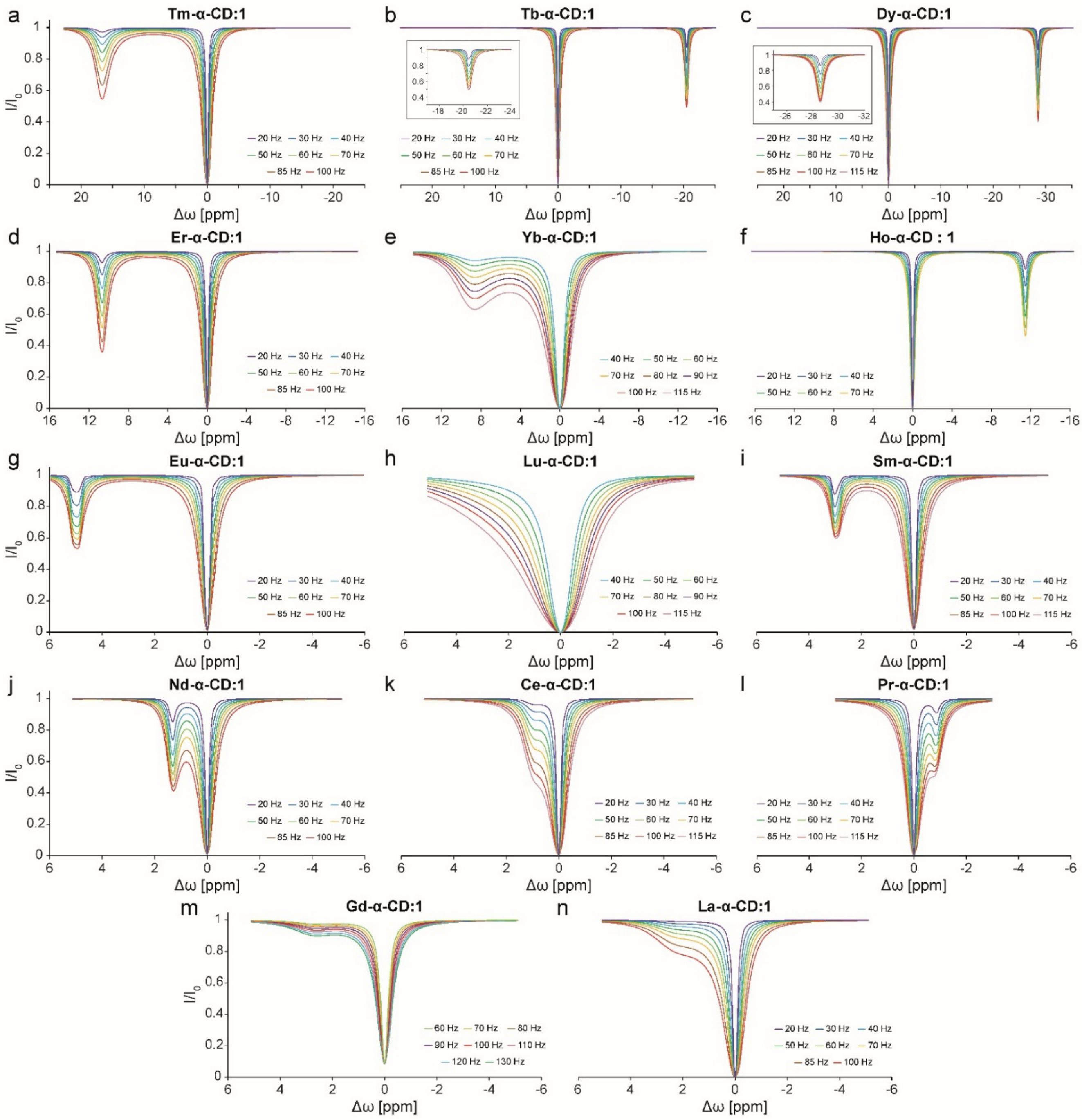

Figure S4. ^{19}F-ParaGEST Characterization. Multipower ^{19}F-ParaGEST z-spectra fittings for samples containing Ln-α-CD and guest **1** in a 1:200 ratio. Ln = (a) Thulium; (b) Terbium; (c) Dysprosium; (d) Erbium; (e) Ytterbium; (f) Holmium; (g) Europium; (h) Lutetium; (i) Samarium; (j) Neodymium; (k) Cerium; (l) Praseodymium; (m) Gadolinium and (n) Lanthanum.

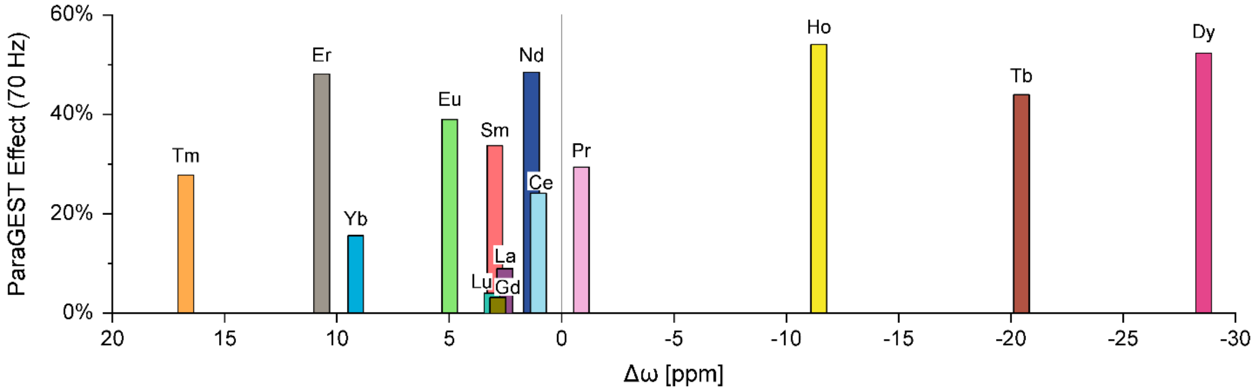

Figure S5. ^{19}F-ParaGEST Effects Library. Bar graph representations of the chemical shift offset (Δω) and the size of the effect (at 70 Hz saturation) for each Ln-α-CD:**1** pair.

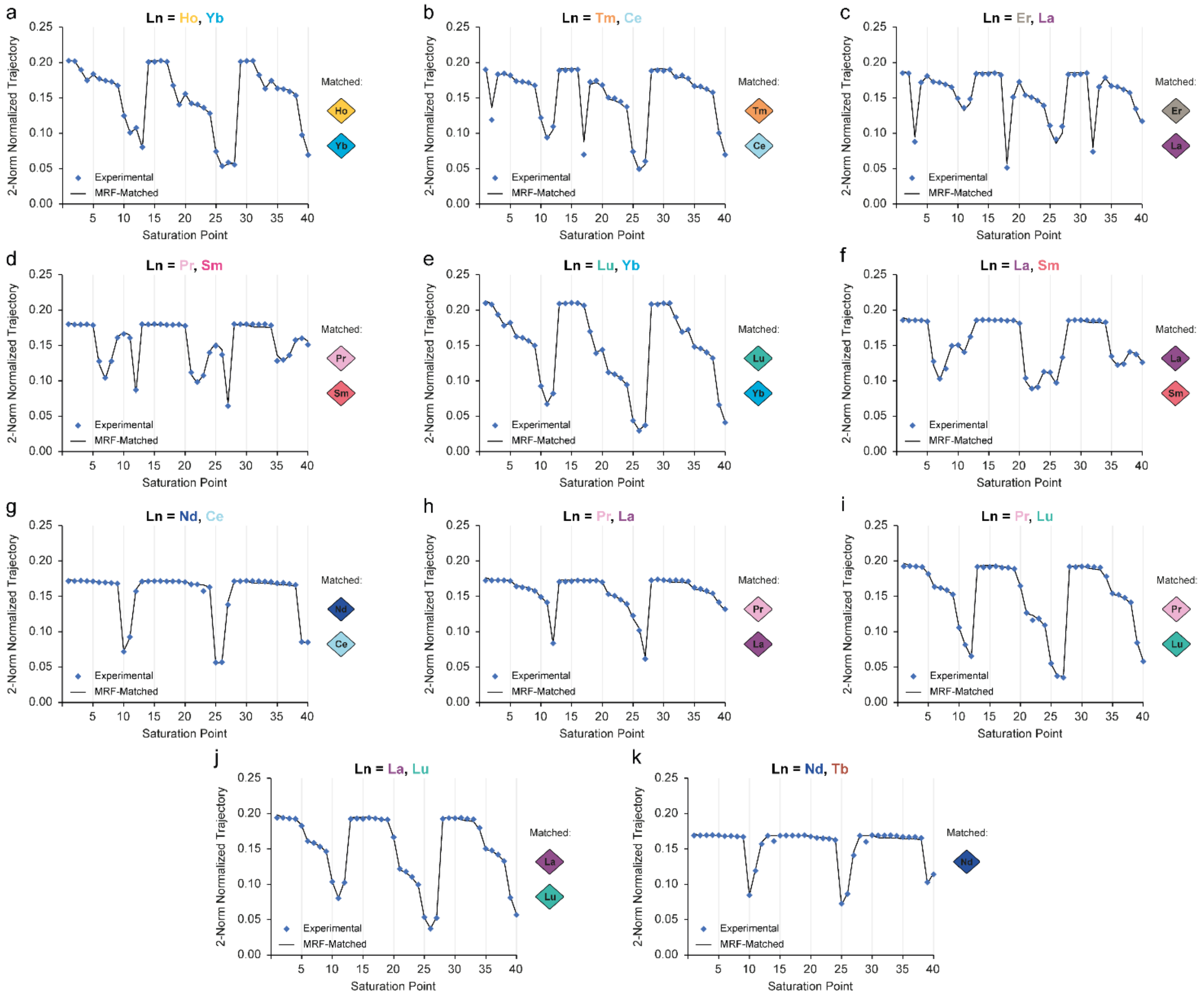

Figure S6. ^{19}F-ParaGEST Fingerprint Analysis of Samples Containing Two Lanthanide Mixtures. Experimental (colored diamonds) and matched (black line) ^{19}F-ParaGEST fingerprints for samples containing Ln-α-CDs and guest 1 in a 1:1:200 ratio. Ln = (a) Holmium and Ytterbium; (b) Thulium and Cerium; (c) Erbium and Lanthanum; (d) Praseodymium and Samarium; (e) Lutetium and Ytterbium; (f) Lanthanum and Samarium; (g) Neodymium and Cerium; (h) Praseodymium and Lanthanum; (i) Praseodymium and Lutetium; (j) Lanthanum and Lutetium; (k) Neodymium and Terbium. Experimental data was collected in a 11.75T NMR scanner. The lanthanides extracted from each matched fingerprint are represented in diamonds.

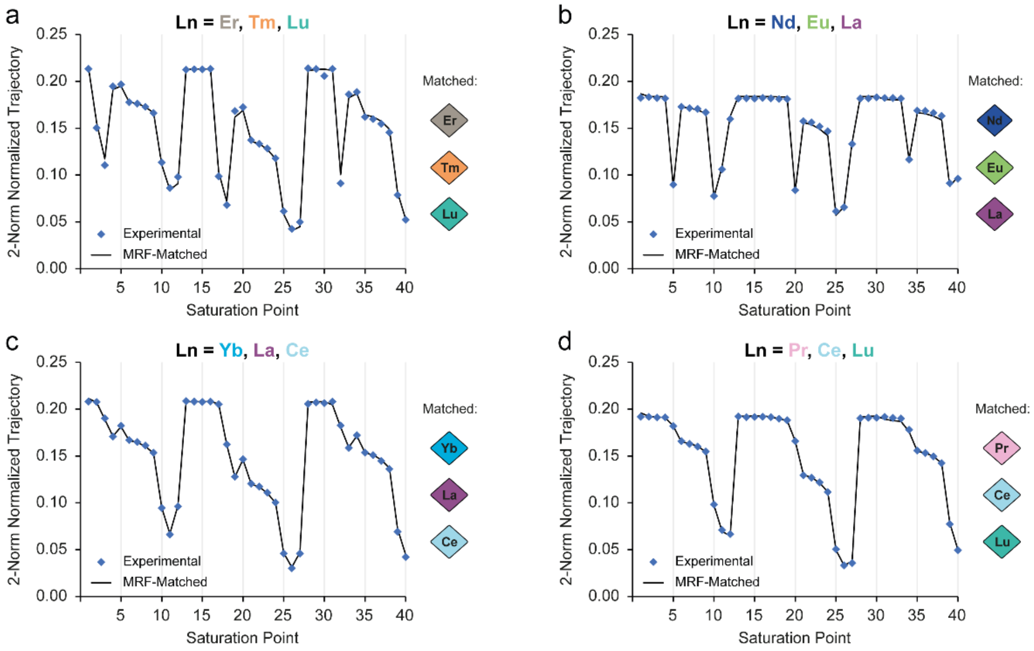

Figure S7. ^{19}F-ParaGEST Fingerprint Analysis of Samples Containing Three Lanthanide Mixtures. Experimental (colored diamonds) and matched (black line) ^{19}F-ParaGEST fingerprints for samples containing Ln-α-CDs and guest 1 in a 1:1:1:200 ratio. Ln = (a) Erbium, Thulium and Lutetium; (b) Neodymium, Europium and Lanthanum; (c) Ytterbium, Lanthanum and Cerium; (d) Praseodymium, Cerium and Lutetium. Experimental data was collected in a 11.75T NMR scanner. The lanthanides extracted from each matched fingerprint are represented in diamonds.

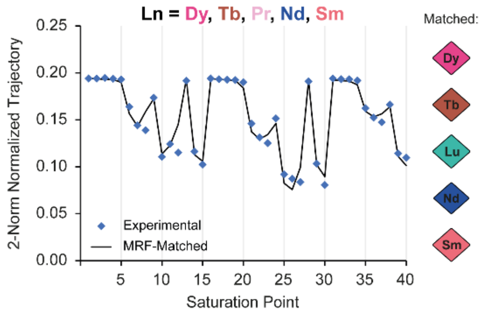

Figure S8. ^{19}F-ParaGEST Fingerprint Analysis of Samples Containing Five Lanthanide Mixtures. Experimental (colored diamonds) and matched (black line) ^{19}F-ParaGEST fingerprints for samples containing Ln-α-CDs and guest 1 in a 1:1:1:1:1:200 ratio. Ln = Dysprosium, Terbium, Praseodymium, Neodymium and Samarium; Experimental data was collected in a 11.75T NMR scanner. The lanthanides extracted from each matched fingerprint are represented in diamonds.

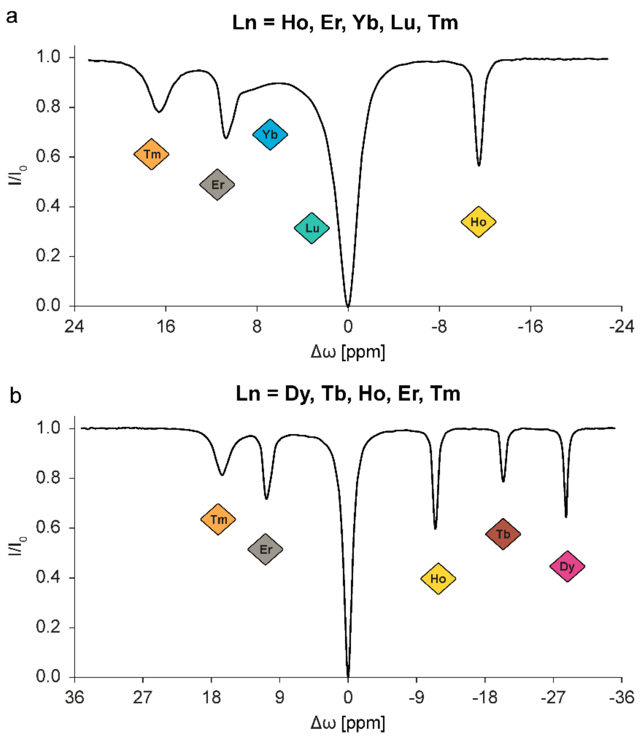

Figure S9. ^{19}F-ParaGEST fingerprints for samples containing Ln-α-CDs and guest 1 in a 1:1:1:1:1:200 ratio. Ln = (a) Holmium, Erbium, Ytterbium, Lutetium and Thulium (Fig. 4h); (b) Dysprosium, Terbium, Holmium, Erbium and Thulium (Fig. 4i). Experimental data was collected in a 11.75T NMR scanner. The assignment of each effect is represented in a diamond.

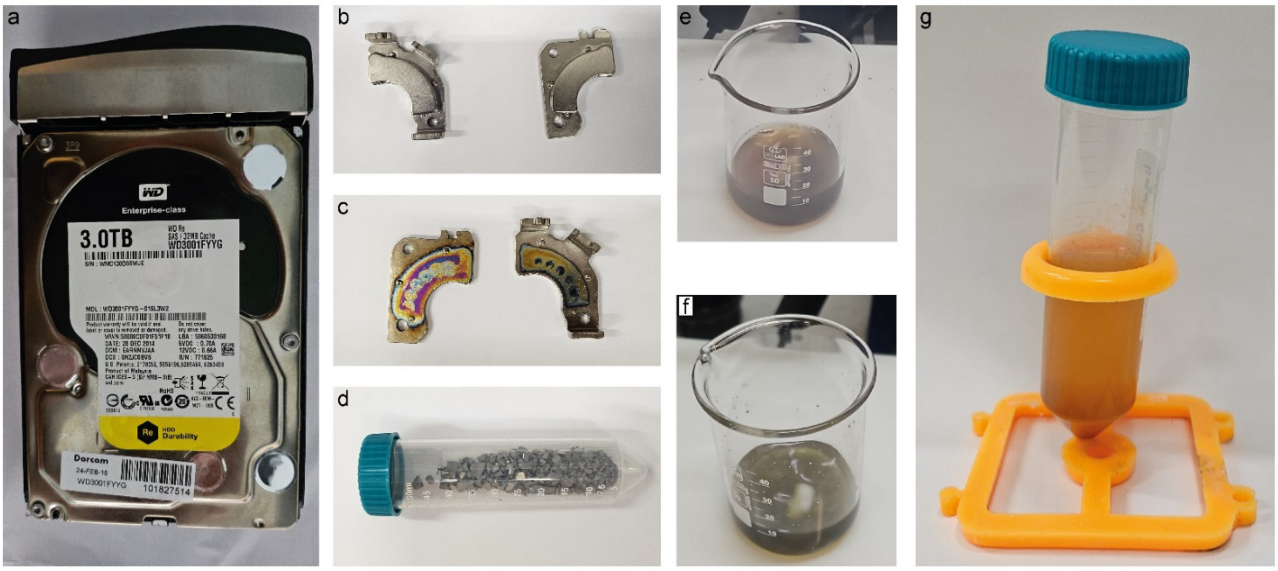

Figure S10. Lanthanide extraction from Hard Disk Drive (HDD). (a) Western Digital Re WD3001FYYG 3TB Hard disk drive; (b) Isolated NdFeB magnets inside metal frames; (c) Metal frames after the removal of demagnetized magnets; (d) Crushed demagnetized NdFeB magnets; (e) Acid leaching of lanthanides from crushed magnet pieces; (f) Final appearance of the leaching solution; (g) Final unknown solution after filtration on titration to pH 2-3.

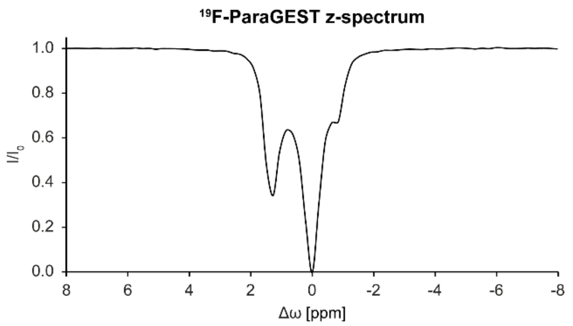

Figure S11. **^{19}F-ParaGEST z-spectra collected for HDD extract with α-CD-DTPA and 1 (unknown solution).**

Supporting Tables

Table S1. **^{19}F-ParaGEST parameters for Ln-α-CD:1 pairs in 1:200 ratio.** T_1 and T_2 = longitudinal and transverse relaxation rates of the free guest in presence of the Ln-α-CD; Δω = ^{19}F-ParaGEST effect's chemical shift offset; f_B = bound fraction of host-guest complex; k_{ex} = exchange rate.

Ln-α-CD	pH	T_1 [sec]	T_2 [msec]	Δω [ppm]	f_B	k_{ex} [Hz]
**Dy**	10.10	2.39	400.00	-28.58	2.22E-03	336.95
**Tb**	10.04	2.59	666.00	-20.47	2.08E-03	241.42
**Ho**	10.12	2.34	445.00	-11.45	2.42E-03	417.03
**Pr**	10.05	3.22	915.70	-0.88	1.26E-03	278.04
**Nd**	10.07	3.14	882.60	1.34	1.96E-03	321.04
**Eu**	10.11	3.16	898.00	4.99	2.78E-03	154.25
**Yb**	10.09	3.14	64.08	9.17	2.81E-03	6063.51
**Er**	10.07	2.39	221.00	10.69	2.94E-03	1056.47
**Tm**	10.05	2.75	111.99	16.73	2.74E-03	3076.54
**La**	10.08	3.23	460.50	2.53	8.24E-04	2778.15
**Ce**	10.05	3.22	765.60	1.04	1.07E-03	812.49
**Sm**	10.10	3.17	942.60	2.98	1.70E-03	179.66
**Gd**	10.07	1.14	598.80	2.84	2.17E-04	1326.61
**Lu**	10.06	3.22	69.17	3.10	3.44E-03	8169.28

Table S2. Chemical Shift Offsets (Δω) for ^{19}F-ParaGEST MRF Acquisition Protocol.

Saturation Point	Δω [ppm]	Expected Ln
1	300.0	-
2	16.7	Tm
3	10.7	Er
4	9.2	Yb
5	5.0	Eu
6	3.1	Lu
7	3.0	Sm
8	2.8	Gd
9	2.5	La
10	1.3	Nd
11	1.0	Ce
12	-0.9	Pr
13	-11.5	Ho
14	-20.5	Tb
15	-28.6	Dy
16	300	-
17	16.7	Tm
18	10.7	Er
19	9.2	Yb
20	5.0	Eu
21	3.1	Lu
22	3.0	Sm
23	2.8	Gd
24	2.5	La
25	1.3	Nd
26	1.0	Ce
27	-0.9	Pr
28	-11.5	Ho
29	-20.5	Tb
30	-28.6	Dy
31	300.0	-
32	10.7	Er
33	9.2	Yb
34	5.0	Eu
35	3.1	Lu
36	3.0	Sm
37	2.8	Gd
38	2.5	La
39	1.3	Nd
40	1.0	Ce

Table S3. Concentrations of Ln-α-CDs used for generating dictionary entries. Values were selected based on experimental sample preparation and the volume fractions extracted in Table S1.

	Min Conc.	Interval	Max Conc.	No. of Combinations	No. Entries Per Ln (determined by the number of possible conc.)	Total No. Entries
**Single Ln**	172 nM	172 nM	129 µM	14	750	10,500
**2 Ln's**	10.32 µM	1.72 µM	103.2 µM	91	55	275,275
**3 Ln's**	10.32 µM	8.6 µM	96.32 µM	364	11	484,484
**5 Ln's**	17.2 µM	17.2 µM	86 µM	2,002	5	6,256,250
**Full Dictionary Specifications**				**2,471**	**821**	**7,026,509**

Table S4. Dot product results for fingerprint assignment of single lanthanide samples.

Figure	Experimental	Matched	Dot Product
**2e**	Yb	Yb	0.999975
**2f**	Dy	Dy	0.99981
**3a**	Tm	Tm	0.99971
**3b**	Tb	Tb	0.999904
**3c**	Er	Er	0.999921
**3d**	Ho	Ho	0.999824
**3e**	Eu	Eu	0.999885
**3f**	Lu	Lu	0.999967
**3g**	Sm	Sm	0.999941
**3h**	Nd	Nd	0.99994
**3i**	Ce	Ce	0.999953
**3j**	Pr	Pr	0.999945
**3k**	Gd	Gd + Pr	0.999891
**3l**	La	La	0.999956

Table S5. Dot product results for fingerprint assignment of two lanthanides samples.

Figure	Ln 1	Ln 2	Matched	Dot Product
**4b**	Tb	Eu	Tb + Eu	0.999927
**4c**	Dy	Nd	Dy + Nd	0.999967
**S6a**	Ho	Yb	Ho + Yb	0.99997
**S6b**	Tm	Ce	Tm + Ce	0.999596
**S6c**	Er	La	Er + La	0.999795
**S6d**	Pr	Sm	Pr + Sm	0.999839
**S6e**	Lu	Yb	Lu + Yb	0.999972
**S6f**	La	Sm	La + Sm	0.999891
**S6g**	Nd	Ce	Nd + Ce	0.99992
**S6h**	Pr	La	Pr + La	0.999938
**S6i**	Pr	Lu	Pr + Lu	0.999929
**S6j**	La	Lu	La + Lu	0.999954
**S6k**	Nd	Tb	Nd	0.999867

Table S6. Dot product results for fingerprint assignment of three lanthanides samples.

Figure	Ln 1	Ln 2	Ln 3	Matched	Dot Product	Notes
**4e**	Dy	Pr	Nd	Dy + Pr + Nd	0.999922	
**4f**	Eu	Ce	Sm	Eu + Ce + Sm	0.999533	
**S7a**	Er	Tm	Lu	Er + Tm + Lu	0.999759	
**S7b**	Nd	Eu	La	Nd + Eu+ La	0.99989	
**S7c**	Yb	La	Ce	Yb + La + Ce	0.999968	
**S7d**	Pr	Ce	Lu	Pr + Ce + Lu	0.999963	

Table S7. Dot product results for fingerprint assignment of five lanthanides samples.

Figure	Ln 1	Ln 2	Ln 3	Ln 4	Ln 5	Matched	Dot Product
**4h**	Ho	Er	Yb	Lu	Tm	Ho + Er + Yb + Lu + Tm	0.999332
**4i**	Dy	Tb	Ho	Er	Tm	Dy + Tb + Ho + Er + Tm	0.999183
**S9**	Dy	Tb	Pr	Nd	Sm	Dy + Tb + Lu + Nd + Sm	0.998805

Table S8. ICPMS measurement of lanthanide content in hard disk drive extract. Final concentrations were calculated from averaging the readings of two dilutions (1/100,000 and 1/5,000). BLQ = below derection limit (1 ppb).

Ln Isotope	Final C. [ppb]	Final C. [mM]
**^{139} La [He]**	BLQ	**-**
**^{140}Ce [He]**	BLQ	**-**
**^{146}Nd [He]**	367.95	**26.80**
**^{153}Eu [He]**	BLQ	**-**
**^{159}Tb [He]**	8.05	0.54
**^{163}Dy [He]**	0.9	0.03
**^{89}Y [He]**	BLQ	-
**^{141}Pr [He]**	102.7	**7.98**
**^{147}Sm [He]**	BLQ	-
**^{157}Gd [He]**	3.7	0.26
**^{165}Ho [He]**	BLQ	-
**^{166}Er [He]**	0.8	0.02
**^{169}Tm [He]**	BLQ	-
**^{172}Yb [He]**	BLQ	-
**^{175}Lu [He]**	BLQ	-

Supporting References

(1) Zaiss, M.; Angelovski, G.; Demetriou, E.; McMahon, M. T.; Golay, X.; Scheffler, K. *Magn. Reson. Med.* **2018**, *79* (3), 1708-1721.

(2) Zaiss, M.; Bachert, P. *NMR Biomed.* **2013**, *26* (5), 507-518.

(3) Zaiss, M.; Zu, Z.; Xu, J.; Schuenke, P.; Gochberg, D. F.; Gore, J. C.; Ladd, M. E.; Bachert, P. *NMR Biomed.* **2015**, *28* (2), 217-230.

(4) Vladimirov, N.; Cohen, O.; Heo, H.-Y.; Zaiss, M.; Farrar, C.; Perlman, O. *Nat. Protoc.* **2025**.

(5) Perlman, O.; Herz, K.; Zaiss, M.; Cohen, O.; Rosen, M. S.; Farrar, C. T. *Magn. Reson. Med.* **2020**, *83* (2), 462-478. From NLM.

(6) Ma, D.; Gulani, V.; Seiberlich, N.; Liu, K.; Sunshine, J. L.; Duerk, J. L.; Griswold, M. A. *Nature* **2013**, *495* (7440), 187-192.

(7) Bahl, C. R. H.; Eder, M. A.; Boland, G.; Abrahamsen, A. B. *IEEE Trans. Magn.* **2020**, *56* (8), 1-6.

(8) Rabatho, J. P.; Tongamp, W.; Takasaki, Y.; Haga, K.; Shibayama, A. *J. Mater. Cycles Waste Manage.* **2013**, *15* (2), 171-178.
